# Circulating Levels of Visceral Adipose Tissue-Derived Serine Protease Inhibitor (Vaspin) Appear as a Marker of Musculoskeletal Pain Disability

**DOI:** 10.3390/diagnostics10100797

**Published:** 2020-10-08

**Authors:** Nader Tarabeih, Alexander Kalinkovich, Adel Shalata, Gregory Livshits

**Affiliations:** 1 Department of Anatomy and Anthropology, Sackler Faculty of Medicine, Tel-Aviv University, Tel-Aviv 6905126, Israel; nadertar@gmail.com (N.T.); alexander.kalinkovich@gmail.com (A.K.); 2 The Simon Winter Institute for Human Genetics, Bnai Zion Medical Center, The Ruth and Bruce Rappaport Faculty of Medicine, Technion, Haifa 32000, Israel; adel.shalata@gmail.com; 3 Adelson School of Medicine, Ariel University, Ariel 4077625, Israel

**Keywords:** low back pain, disability, body composition, vaspin, extracellular water

## Abstract

Musculoskeletal pain (MSP), specifically low back pain (LBP), is often associated with several adipose tissue-derived cytokines (adipokines) and body composition, but their correlations with the LBP-related disability/severity phenotypes remain poorly understood. In this cross-sectional study, two self-reported validated questionnaires were used to collect back pain and disability data in an ethnically homogeneous family-based population sample (*N* = 1078). Plasma levels of relatively new adipokines, vaspin and adipsin, were detected by ELISA. Body composition parameters, including fat, skeletal muscle mass, extracellular water (ECW), and others were assessed through bioelectrical impedance analysis (BIA) technology. Statistical analysis was conducted, accounting for the familial composition of the sample. The multiple regression analyses with four LBP-related phenotypes as dependent variables consistently showed, for the first time, the significant associations with vaspin levels, regardless of other covariates. The odds ratios (OR)/SD ranged between 1.24 (95%CI = 1.03–1.50) and 1.33 (95%CI = 1.07–1.64), depending on the LBP phenotype. Among the tested body composition covariates, only ECW levels displayed consistent and highly significant associations with all tested LBP phenotypes (OR from 1.43, 95%CI = 1.14–1.79 to 1.68, 95%CI = 1.26–2.24). The results clearly suggest that circulating concentrations of vaspin and ECW levels could serve as biomarkers of MSP/LBP severity and complications.

## 1. Introduction

Pain is commonly defined as an unpleasant sensation or physical suffering caused by trauma or illness. It is often accompanied by anatomical and/or physiological alterations, which, however, frequently remain uncertain [1]. Therefore, an identification of the reliable biomarkers of pain would have obvious therapeutic and preventive medicine significance. One of the most common pain symptoms experienced by people of all ages is musculoskeletal pain (MSP), in particular low back pain (LBP). LBP is also the number one cause of disability globally affecting >500 million people at any given time [2]. However, its etiology and pathogenesis are largely unknown. In particular, the mechanisms determining the transition from acute to chronic stage, intensity of the disease, and the response to specific therapies are still indeterminate. However, most importantly, there have been no validated biomarkers found that could enhance our understanding of the mechanisms of LBP pathophysiology. Previous studies have reported a close association of LBP with sarcopenia of the paraspinal muscles [3,4,5], suggesting that skeletal muscle-associated molecules might serve as LBP biomarkers. Indeed, we have recently reported a significant association between the circulating levels of growth and differentiation factor 15 (GDF-15) and LBP disability in a large community-based sample [6]. Taking into account proposed involvement of inflammation in intervertebral disc (IVD) degeneration [7,8,9,10], the search for peripheral biomarkers has been conducted among pro-inflammatory factors that revealed, however, the inconsistent results [11,12]. Although controversial, other studies have suggested obesity as a risk factor in the development of LBP and other MSP syndromes [13,14,15]. In obesity, adipose tissue (AT) releases a great variety of bioactive molecules, specifically adipokines. They have been consistently reported to play an important role in several pain conditions, including chronic widespread pain [16], osteoarthritis (OA) [17,18], bone pathology, and intervertebral disc (IVD) degradation (IVDD) [19,20]. However, in LBP, the attempts to use adipokines, such as adiponectin and leptin, as biomarkers revealed inconsistent results [21,22].

Recently, adipokine adipsin, presumably involved in OA pathogenesis [23], has been proposed as a LBP biomarker [24]. However, this conclusion was drawn from the single study testing a relatively small sample of both affected and control individuals (*n* = 62). A newly discovered adipokine, visceral adipose tissue-derived serine protease inhibitor (vaspin, SERPINA12) was found to be associated with obesity in human subjects [25,26,27,28]. Moreover, vaspin is produced by skeletal muscle, and apparently is involved in bone metabolism in an obesity-dependent manner [28,29]. However, no studies have explored vaspin in relation to LBP.

Association of MSP manifestations with body composition characteristics were also repeatedly reported [30,31,32,33]. Body mass index (BMI) and other anthropometric measures of obesity were found to be associated with LBP intensity in both men and women [34,35,36], suggesting the involvement of AT in LBP pathogenesis. However, in these studies, the peripheral levels of adipokines that can potentially serve as the peripheral molecular LBP biomarkers, have not been assessed. Therefore, in search for such biomarkers, the present study aimed to examine the possible combined effect (association) of the plasma levels of vaspin and adipsin, on the one hand, and body composition components, on the other hand, on the LBP-related disability in a large, family-based population sample.

## 2. Materials and Methods

### 2.1. Sample

The data were collected in outpatient clinics located in Sakhnin, from January 2014 to January 2016, focusing on an ethnically Arab population in Israel, characterized by a stable family structure, traditional relationships, similar shared living, economic, and professional conditions, with access to modern medical facilities. It is noteworthy that this population was with high prevalence of LBP in people of relatively young age (<45) [37]. The families were selected via a proband (<50 years of age) who was previously diagnosed with LBP by a physician, confirmed by an orthopedist, and had at least one first-degree relative diagnosed with a similar LBP condition. The unaffected members (with no LBP symptomatology) from the same family were considered as the control group. Assessment of the families was performed in accordance with our detailed protocol and conducted by certified and experienced nurses as follows.

Each individual, regardless of their LBP status, completed two questionnaires, which have been implemented in many other studies on this subject. The questionnaires were translated into Arabic and validated. Demographic data, anthropometric measurements, body composition, and blood sample were collected. The blood samples were used to assay the plasma concentrations of the biochemical factors relevant to this study. Data were collected from 1078 individuals belonging to 98 nuclear families, with one to eleven siblings per family. A total of 26 of the recruited individuals were excluded due to severe heart problems, pregnancy, and/or for being under 16 years of age. Each participant signed an informed consent form written in Arabic prior to their participation and data collection. This research was approved by the IRB-Helsinki Committee (Number: 2013/042K, Date: 04.11.2013) of the Meir Medical Center, Kfar Saba, Israel, and the Ethics Committee of Tel Aviv University, Tel Aviv, Israel.

### 2.2. LBP-Phenotypes

Since LBP is a heterogeneous clinical condition, different etiological factors may contribute to its disease manifestation and severity. In attempt to cover the variety of the disease manifestations and accepted clinical assessments, LBP was assessed by an orthopedic physician and two self-reported questionnaires: the Medical Research Council Nurses’ Study questionnaire (MRCQ) and the Roland-Morris Disability Questionnaire (RMDQ) [38,39]. Our analysis focused on three primary binary phenotypes: (1) LBP-sciatica—patients with sciatica were identified by the physician, defined as yes vs. no. The following two phenotypes were determined based on the MRCQ: (2) LBP-duration, defined as “the duration of pain felt during the past 10 years, more so than 12 months altogether”; (3) LBP-severity, defined as “During the past 12 months, have you had to take time off from work for at least one week due to your low back pain?” (yes/no); the fourth LBP phenotype, LBP-disability, was based on the attained RMDQ scores. RMDQ is a complementary approach in assessing physical disability in patients with LBP, and has been shown to yield reliable measurements in determining the level of disability [39]. This quantitative measure comprises 24 items specifically related to physical functions most likely to be affected by LBP. Each item is qualified with the phrase “because of my back pain” to distinguish back pain disability from disability due to other causes [40]. The RMDQ score was calculated by summing up the number of items checked. Items were unweighted. The scores ranged on a scale from 0–24, with greater levels of disability reflected by higher numbers.

### 2.3. Demographic, Anthropometric, and Body Composition Assessment

A trained research nurse assessed data. Demographic data included age and gender. Elsewhere [6], we recently described in detail anthropometric measurements and body composition assessment using bio-impedance (BIA) technology. Briefly, in the present study, we used anthropometrically measured body mass index (BMI) in kg/m^2^ and waist-to-hip ratio (WHR) in mm/mm. Body composition measures assessed by BIA included fat mass (FM), skeletal muscle mass (SMM), and body-cell mass (BCM), all in kilograms; extracellular water (ECW), in liters. As body mass components strongly intercorrelated and depend on body weight, they were examined as indices; i.e., FM/WT, SMM/WT, and BCM/WT. ECW has important physiological significance, as >50% of the body weight is water, distributed between intracellular and extracellular components. The body’s homeostatic physiological mechanisms maintain ECW [41], and deviation from this equilibrium could cause, for example, an edematous state; or loss of water can lead to body dehydration. Therefore, these measures may reflect a clinical significance of the present study.

### 2.4. Soluble Biomarker Analysis

Venous blood samples measured by a venipuncture following overnight fasting underwent centrifugation within 1h after collection, according to the standard protocol. Plasma samples were separated and stored in aliquots at −80 °C until usage. Circulating levels of vaspin and adipsin were detected by ELISA using DuoSet kits (R&D Systems, Minneapolis, MN, USA), according to the manufacturer’s protocols. The detection limits were 49.6 pg/mL for vaspin and 375 µg/mL for adipsin. The intra- and inter-assay coefficients of variation were between 2.3% and 8.3%. Due to the significant deviation of the respective distribution from the normality assumptions, the original measurements of these factors were subjected to a log-normal transformation to approximate normality prior to analysis.

### 2.5. Statistical Analysis

Using standard statistical packages SPSS (IBM SPSS Statistics for Windows, Version 24.0. Armonk, NY, USA: IBM Corp) and Statistica 64 (TIBCO Software, Version 13.5, Palo Aalto, CA, USA), basic descriptive statistics were computed, and the normality assumptions of the distribution of the study’s quantitative variables were tested. This stage included identification of outliers and the selection of potential covariates for LBP-related phenotypes, such as sex, age, and body composition, as well as implementation of correlation/regression analyses and ANOVAs. The obtained results are summarized in the Appendix A (Table A1, Table A2 and Table A3).

Our sample comprised 98 nuclear families; therefore, the parameter estimates obtained by the standard methods of the statistical analysis could be biased. To overcome this problem, we implemented mixed statistical models with a flexible kinship covariance structure appropriate for the samples including individuals with multiple levels of relatedness. We used the Kinship Statistical Package for R (https://github.com/cran/kinship/blob/master/R/lmekin.R, Access date: 20.07.2020). The *relmatGlmer* function was used to assess logistic regression mixed models (for binary outcomes), and the *relmatLmer*, similar to the *relmatGlmer*, was used to generate a linear regression mixed model (for continuous traits). This process was implemented at the final stage of the analysis of each of the LBP-related phenotypes in order to accurately establish their possible relationships with the contributing soluble markers and body composition variables.

Once biochemical and body composition variables associating significantly with the LBP-phenotypes were detected, we attempted to estimate contribution of the putative genetic factors to variation of each of them. We also attempted to evaluate whether, and to what extent (if any), the aforementioned significant associations could be caused by shared genetic factors. To this aim, we conducted family-based variance (VCA, for continuous variables) and quasi-variance (Q_VCA, for binary phenotypes) decomposition analyses, described in detail elsewhere [42,43]. This method, implemented in the MAN statistical package (https://www.tau.ac.il/~idak/MAN_Manual.pdf, Access date: 20.07.2020), decomposes the total phenotype variation into components presumably caused by additive genetic factors, common family environment, and the residual component of the variance. Similarly, the bivariate version of the analysis examines the contribution of the same factors (putative genetic and shared familial environment) to the phenotypic correlation/association between the phenotypes. The MAN package uses a standard hierarchical (nested) testing of the statistical-genetic models, comparing them through a likelihood ratio test (LRT), and selecting the best fitting and most parsimonious model.

## 3. Results

### 3.1. Sample Characteristics

The descriptive statistics and ranges of variations in all examined anthropometric body composition variables and levels of the circulating adipokines are presented in Table A1 (separately comparing males and females). In total, 489 males and 589 females participated in this study and displayed non-significant (*p* = 0.65) age differences between the sexes (42.80 ± 0.62 vs. 43.20 ± 0.56 years, respectively). However, body composition variables, as expected, were significantly different, with BMI greater in females (28.72 ± 0.24 vs. 27.53 ± 0.19, *p* < 0.0001), and WHR, an indicator of central obesity, greater in males (0.92 ± 0.003 vs. 0.88 ± 0.004, *p* < 0.001). Females compared to males had a higher FM/WT (0.36 ± 0.003 vs. 0.25 ± 0.002, respectively, *p* < 0.001) and lower muscle mass measurements (such as SMM/WT: 0.27 ± 0.001 vs. 0.37 ± 0.002, respectively, *p* < 0.001), while males had a higher ECW compared to females (20.75 ± 0.15 vs. 16.53 ± 0.10, respectively, *p* < 0.001) (Table A1).

The levels of vaspin (pg/mL) were significantly higher in females compared to males (1605.21 ± 210.53 vs. 1603.59 ± 233.68, respectively, *p* = 0.001); yet there was no difference between males and females in adipsin (µg/mL) levels (1.30 ± 0.01 vs. 1.28 ± 0.01, respectively, *p* = 0.054). In the one-sided t-test, the prevalence of LBP-sciatica and LBP-duration was significantly more common in women than in men [45.50% (*N* = 589) among women vs. 37.20% (*N* = 489) among men, *p* < 0.001 and 33% (195/589) among women vs. 27% (132/489) among men, *p* = 0.008], respectively. The LBP-severity phenotype was found in 195 males (40%) and 150 females (25.50%), *p* < 0.0001.

The variations of the studied phenotypes were not entirely independent (Table A2). Obesity indicators (BMI and WHR) and adipsin in males and females exhibited significant, albeit low-magnitude, positive correlations (r ranged from 0.12 to 0.35, *p* < 0.001). Correspondingly, skeletal muscle mass measurements (SMM/WT and BCM/WT) displayed a significant negative correlation with adipsin (r ranged between −0.16 and −0.30, *p* < 0.001). In males, vaspin revealed a significant negative correlation with BCM/WT (r = −0.10, *p* < 0.05), in addition to significant positive correlations with BMI (r = 0.11, *p* < 0.05) and ECW (r = 0.14, *p* < 0.01) (Table A2).

Body composition variables were also significantly intercorrelated, in particular BMI, WHR, and ECW as well as FM/WT, SMM/WT, and BCM/MT (Table A2). Since SMM/WT and FM/WT were highly collinear with BCM/WT (r ranged from −0.83 to 0.75, *p* < 0.001), in the following analysis we used only BCM/WT to avoid redundancy and multicollinearity in multiple regression analysis.

### 3.2. Associations between LBP-Related Phenotypes and Covariates

As shown in Table 1, in the series of univariate analyses, individuals who reported LBP-sciatica exhibited higher ECW and obesity measures (WHR and lower BCM/WT) than those with no LBP symptoms (controls), even after controlling for sex and age. The plasma concentrations of vaspin and adipsin, in addition to ECW, tended to be significantly higher in patients with LBP-sciatica, however, only vaspin and ECW remained significant after adjustment. Worsening of the LBP manifestations, including LBP-duration, and LBP-severity, were all significantly associated with elevation in vaspin and adipsin plasma levels, increased ECW, and adiposity characteristics (WHR), and correspondingly reduced skeletal muscle mass measure (BCM/WT). However, adipsin levels were not significant after adjustment for sex and age. Significant correlations were observed between body composition measures (BCM/WT and ECW) and vaspin circulating levels with LBP-disability scores in both males and females based on the RMDQ (Table 2). No significant sex-associated differences in vaspin and adipsin levels in LBP-related phenotypes were observed (Table A3).

### 3.3. Mixed Model-Multivariable Analysis of LBP-Related Phenotypes

At this stage, all potential predictor variables (covariates) that were significantly associated with LBP-related phenotypes in the univariate context (Table 1 and Table 2) were analyzed by a mixed logistic regression to simultaneously test the extent of the associations of the body composition measurements and plasma levels of soluble markers. Since our sample was composed of nuclear families and interrelated complex pedigrees, the corresponding mixed-model linear regressions were employed to account for familial size and structure (Table 3 and Table 4). The results consistently showed that age, sex, ECW, and vaspin levels were independently and significantly associated with all the LBP-related phenotypes.

Thus, the multiple logistic regression analyses produced the odds ratios (OR), reflecting independent association of vaspin levels ranging between 1.24 (95%CI = 1.03–1.50) for LBP-duration and 1.33 (95%CI = 1.07–1.64) for LBP-severity. The OR estimates obtained in the respective multiple logistic regression analyses for ECW effect ranged from 1.43 (95%CI = 1.14–1.79) for LBP-sciatica to 1.68 (95%CI = 1.26–2.24) for LBP-severity (Table 3).

Subsequently, we conducted a mixed multivariable linear regression analysis to identify significant covariates of LBP-disability, assessed by the RMDQ quantitative scores. The design of the analysis (Table 4) was similar to the above (Table 3) and retained the same covariates. Specifically, we found a significant independent association of the vaspin (*p* = 7.42 × 10^−3^**)** and ECW (*p* = 4.44 × 10^−3^) levels with LBP-disability—both types of multivariable regression analyses consistently showed significant associations of the vaspin plasma levels and ECW measures with all tested LBP-related characteristics.

### 3.4. Contribution of Putative Genetic Factors to Significant Associations

The primary LBP-related phenotypes (LBP-sciatica, LBP-duration and LBP-severity) and LBP-disability scores, which showed significant correlations with the vaspin levels and ECW scores, were subjected to corresponding VCA and Q_VCA, as described in the Material and Methods section. To compare the general model with restricted models, we implemented maximum likelihood ratio tests. Our respective most parsimonious models estimated that vaspin levels, ECW, and the LBP-disability variables displayed a significant additive genetic variance component in their interindividual variation, while the other variables displayed no significant component (Table 5). Since our analysis detected a significant genetic component in the LBP-disability scores on the one hand, and in vaspin and ECW interindividual variation on the other, we conducted a bivariate variance component analysis to examine the extent to which the associations reported in Table 3 and Table 4 could be attributable to common genetic and/or environmental factors. These analyses revealed highly significant additive genetic correlation between the LBP-disability scores and vaspin levels (0.631 ± 0.094, *p* ≤ 10^−8^), as well as between ECW and LBP-disability scores (0.485 ± 0.109, *p* ≤ 10^−11^) (data not shown), thus suggesting that shared genetic factors influence variations of the LBP-disability, vaspin levels, and ECW.

## 4. Discussion

This cross-sectional study in symptomatic chronic LBP patients provides interesting insight into the potential roles played by plasma vaspin levels and ECW in LBP complication manifestations and progression. This is probably the first study to report consistent significant associations between vaspin circulating levels and LBP, the most frequent MSP condition. As LBP is a heterogeneous condition [44], we intended to minimize etiological heterogeneity by including into the analysis a sample of individuals from a very similar (virtually identical) ethnic, cultural, and socioeconomic background. Since the chosen variables were all LBP characteristics, they were not completely independent, but some associations were pretty low. Moreover, the LBP manifestations were assessed by two independent questionnaires, and we contrasted control with most severe manifestation of the given phenotype, except LBP-disability, which behaved as quantitative continuous variable.

The series of univariate analyses that we conducted demonstrated significant associations of vaspin and adipsin levels with the majority of body composition characteristics, which were positively and negatively correlated with adipose and skeletal muscle mass parameters, respectively. These analyses also revealed significant associations of plasma levels of these adipokines with various LBP phenotypes, while in the multiple regression analyses, only vaspin levels and ECW measures were consistently and significantly retained in the final stages. Since the variations of the body composition phenotypes were not independent from each other, and were age-dependent (Table A2), this probably explains why only ECW was retained in the multiple regression analyses.

Vaspin is known mainly for its insulin-sensitizing effects in metabolic syndrome, including obesity [27,45,46]. Animal studies demonstrated that vaspin can reduce body weight, improve the whole-body metabolic status, enhance bone strength and trabecular bone mass in obesity [29], promote osteogenesis [29], and prevent osteoclastogenesis [47]. Moreover, vaspin is produced by skeletal muscle and was shown to prevent insulin resistance in human myotubes [28]. These findings clearly indicate vaspin involvement in bone and muscle physiology. In addition, vaspin gene and protein expression was detected in the cartilage, synovium, and osteophytes from OA patients [48], in which serum vaspin concentration were higher than in the synovial fluid, with no association of both levels with age or BMI. Furthermore, vaspin was found to prevent leptin-induced inflammation and catabolism in chondrocytes and apoptosis in endothelial cells induced by free fatty acids [49,50]. These and other examples of the anti-inflammatory activity of vaspin served as a basis for the formulation of the protective function hypothesis of vaspin [51]. It has been suggested that vaspin plays a protective role in various detrimental, mainly inflammatory conditions [52,53], involved in IVDD [7,9], spinal OA [54], and pain sensitization [55]. Our data on a consistently significant and independent association of elevated vaspin levels with the severity of LBP manifestations may reflect its protective role in LBP.

ECW accounts for about 35% of the total body water. Changes in ECW osmolarity are accompanied by a flow of water and presumably key biochemical factors out of the cells, leading to cell shrinkage, oxidative stress, protein alterations, mitochondrial, DNA damage, and cell cycle arrest, thus rendering cells susceptible to apoptosis [56]. Moreover, water leakage from nucleus pulposus of the IVDs was found to be associated with the enhanced production of pro-inflammatory cytokines, such as TNFα and IL-1β, and activation of proteases, thus leading to the extracellular matrix damage and pain sensitization that characterizes IVDD and LBP [57]. ECW leakage is linked to many chronic inflammatory disorders, including obesity [58], arthritis [59], and sarcopenia [60,61]. It has been also shown that an elevation in ECW levels is independently associated with sarcopenia characteristics, such as muscle strength, functional capacity, gait speed, and frailty [62]. Sarcopenia of paraspinal muscles, in turn, has been found to be correlated with LBP manifestations [3,4,5]. In our study, ECW was consistently independent of the covariates and highly significantly associated with all the primary phenotypes (Table 3 and Table 4), displaying significant negative correlations with the skeletal muscle measurements (SMM/WT and BCM/WT). These findings suggest that elevated ECW levels might indicate the presence of sarcopenia in individuals suffering from LBP. Thus, ECW is an important and relevant biomarker (probably, a risk factor) for LBP-related conditions.

Given the significant associations between the various LBP manifestations and ECW and vaspin levels in our family-based sample, our next research question was: to what extent could these associations be attributable to common genetic factors (pleiotropy)? Or rather, are they caused mostly by environmental factors, such as lifestyle or trauma, for example? Although the role of genetic regulation of LBP manifestations remains unclear, some studies, including our own previous study, suggested the presence of a genetic component governing interindividual variation of LBP, with heritability estimates often >30% [15,42]. In these studies, presence or absence of LBP during a specific period of time was mostly used as primary phenotype. Here, we used a more complex assessment of the LBP-caused disability and severity, and found that most of these indicators, except LBP-disability scores, showed no involvement of the genetic factors to liability of disability manifestations. Our findings, however, demonstrated a significant and substantial genetic effect on interindividual variation of the vaspin plasma levels and ECW. Additive heritability estimates comprised 66.3 ± 8.5% for vaspin levels and 42.0 ± 6.4% for ECW, but modest 19.9 ± 2.4% for LBP-disability scores. Highly significant additive genetic correlations between the vaspin levels and LBP-disability scores (0.631 ± 0.094, *p* ≤ 10^−8^) and between ECW and LBP-disability scores (0.485 ± 0.109, *p* ≤ 10^−11^) suggest that shared genetic factors influence variations of LBP-disability scores and both of ECW and vaspin levels separately. These findings also suggest that the corresponding correlations of vaspin levels and ECW with LBP-disability scores are caused at least partially by common genetic factors, although the magnitude of the effect is small due to the modest contribution of heredity to LBP-disability scores’ variation.

This seems to be the first estimate of the contribution of the putative genetic factors to variation in vaspin circulating levels. However, some evidence of the genetic determination of vaspin levels were previously published. For example, elevated vaspin serum levels observed in 7% of the Japanese population were found to be linked to minor allele in the rs77060950 polymorphism [53]. Moreover, the minor allele in another polymorphism mapped to vaspin’s structural gene, rs2236242, was proposed to play a protective role against obesity and diabetes in Egyptian women [63], whereas in the other studies this vaspin polymorphism was associated with a greater risk of metabolic syndrome [64,65]. Notably, our estimate of a significant additive genetic component in ECW variation, 0.42 ± 0.06, is in agreement with the previously reported heritability estimate, 0.68, observed in the twin study [66]. Higher heritability estimates in twin design studies are quite common.

Contrary to a recent study [24], we were unable to confirm association of adipsin circulating levels with any of our LBP-related phenotypes in multiple regression analyses, despite the fact that in series of univariate analyses its associations with almost all LBP phenotypes were statistically significant. It is quite likely that, due to its significant correlations with age and ECW, especially in women (Table A2), adipsin was not retained in the final regression equation as an independently associated covariate. The previous study used a much smaller sample with a narrow range of ages, and without considering body composition parameters [24]. This difference seems to provide a likely explanation for the discrepancy of the results.

The cross-sectional design introduces some important limitation to this study, as it prevents drawing conclusions about the causality of the associations found. Longitudinal studies are required to establish the cause-and-effect relationships between LBP-related disability and measured body composition variables and soluble factors (especially vaspin and ECW), as well as to evaluate their predictive nature. Obviously, IVD and paraspinal muscle specimens for the measurement of LBP biomarkers would be preferable but are implausible in general population studies. However, determination of serum/plasma levels of various cytokines and other soluble molecules has been used successfully for monitoring the initiation, intensity and progression of LBP, [6,24,67,68,69] and our data confirm these results.

In conclusion, this is the first study providing statistically significant evidence that vaspin circulating concentrations and ECW levels are independently associated with detrimental LBP phenotypes, although the underlying mechanisms of these associations remain unclear. We also found that genetic factors play a significant role in the interindividual variations of both vaspin levels and ECW, but contribute little, if anything, to LBP manifestation phenotypes. These results provide new insights into the metabolic aspects of LBP pathogenesis and highlight the need for larger longitudinal studies to determine whether both vaspin levels and ECW could serve as novel therapeutic targets for monitoring, prevention, and/or treatment of LBP.

## Figures and Tables

**Table 1:** Body composition parameters and plasma levels of soluble markers (covariates) in individuals with LBP-related phenotypes, compared with control subjects.

Covariate	Control (*N* = 472)	LBP-Sciatica (*N* = 447)	P_1_	P_2_	LBP-Duration (*N* = 337)	P_1_	P_2_	LBP-Severity (*N* = 243)	P_1_	P_2_
BMI (kg/m^2^)	27.32 ± 0.231	29.36 ± 0.25	5	NS	29.86 ± 0.30	5	NS	29.77 ± 0.35	5	1
WHR	0.88 ± 0.001	0.91 ± 0.001	5	1	0.92 ± 0.004	5	1	0.93 ± 0.006	5	4
FM/WT	0.30 ± 0.003	0.33 ± 0.004	5	NS	0.34 ± 0.005	5	NS	0.34 ± 0.006	5	NS
SMM/WT	0.33 ± 0.002	0.31 ± 0.003	5	NS	0.30 ± 0.003	5	NS	0.30 ± 0.004	5	NS
BCM/WT	0.36 ± 0.002	0.33 ± 0.003	5	4	0.33 ± 0.004	5	4	0.33 ± 0.004	5	3
ECW (L)	18.03 ± 0.151	19.04 ± 0.171	5	5	19.35 ± 0.212	5	5	19.25 ± 0.262	5	5
Vaspin (pg/mL)	5.83 ± 0.044	6.13 ± 0.063	5	4	6.11 ± 0.074	4	4	6.17 ± 0.090	4	3
Adipsin (µg/mL)	0.20 ± 0.012	0.25 ± 0.012	3	NS	0.26 ± 0.015	4	NS	0.25 ± 0.015	1	NS

Data presented as mean, standard errors; N, sample size; BMI, body mass index; WHR, waist-to-hip ratio; FM/WT, fat mass/weight ratio; SMM/WT, skeletal muscle mass/weight ratio; BCM/WT, body cell mass/weight ratio, ECW, extracellular water; controls, the members of the same families with no LBP manifestations; P_1_ shows significance levels achieved upon comparison of LBP-related phenotypes with control group; P_2_ obtained by ANCOVA controlling for sex and age; 1 ≤ 0.05; 2 ≤ 0.01; 3 ≤ 0.001; 4 ≤ 0.0001; 5 ≤ 0.00001. NS, non-significant, Adipokines’ circulating levels were transformed to approximate normality prior to analysis.

**Table 2:** Pearson correlation between LBP-disability scores and its potential covariates. Correlation coefficients and corresponding *p*-value are shown.

Covariate	Male (*N* = 489)	Female (*N* = 589)
Age (y)	0.226, <0.001	0.294, <0.001
BMI (kg/m^2^)	NS	0.229, <0.001
WHR	0.211, <0.001	0.267, <0.001
FM/WT	NS	0.227, <0.001
SMM/WT	NS	−0.227, <0.001
BCM/WT	−0.149, <0.001	−0.256, <0.001
ECW (L)	0.215, <0.001	0.307, <0.001
Vaspin (pg/mL)	0.123, <0.001	0.125, <0.001
Adipsin (µg/mL)	NS	0.164, <0.001

LBP-disability phenotype assessed by Roland-Morris Disability Questionnaire (RMDQ); N, sample size; BMI, body mass index; WHR, waist-to-hip ratio; FM/WT, fat mass/weight ratio; SMM/WT, skeletal muscle mass/weight ratio; BCM/WT, body cell mass/weight ratio, ECW, extracellular water; NS, non-significant.

**Table 3:** Mixed-effects model logistic regressions analysis exploring relationships between covariates and LBP-related phenotypes.

	LBP-Sciatica			LBP-Duration			LBP-Severity		
Independent	OR (95% CI)	Β (SE)	*p*	OR (95% CI)	Β (SE)	*p*	OR (95% CI)	Β (SE)	*p*
Age	1.78 (1.45–2.18)	0.57 (0.10)	1.92 × 10^−8^	2.17 (1.71–2.76)	0.77 (0.12)	1.29 × 10^−10^	1.87 (1.44–2.73)	0.62 (0.13)	1.87 × 10^−8^
Sex	2.45 (1.60–3.76)	0.89 (0.20)	3.88 × 10^−5^	2.78 (1.72–4.48)	1.02 (0.24)	2.78 × 10^−5^	3.18 (1.81–5.60)	1.15 (0.28)	1.87 × 10^−6^
ECW	1.43 (1.14–1.79)	0.35 (0.11)	0.001	1.59 (1.23–2.05)	0.46 (0.12)	0.0003	1.68 (1.26–2.24)	0.52 (0.14)	0.0003
Vaspin	1.27 (1.07–1.51)	0.24 (0.08)	0.004	1.24 (1.03–1.50)	0.21 (0.09)	0.02	1.33 (1.07–1.64)	0.28 (0.10)	0.008

Data reported as odds ratio with 95% confidence intervals (OR (95% CI)), with corresponding Beta and standard errors B (SE); ECW, extracellular water. Bold *p*-values indicate significant values. At the initial stage of the study, the following independent variables were tested in stepwise-forward manners: age, sex, WHR, BCM/WT, ECW, vaspin. Only statistically significant terms are shown in the table. All quantitative variables were standardized prior to statistical analysis.

**Table 4:** Mixed-effects linear regression analysis exploring relationships between covariates and LBP-disability scores based on RMDQ.

Dependent Variable: LBP-Disability						
Independent	Beta	SE of Beta	B	SE of B	t	*p*-Value
Age	0.245	0.032	0.258	0.034	7.58	7.75 × 10^−8^
Sex	0.210	0.038	0.209	0.038	5.48	5.16 × 10^−14^
ECW	0.185	0.040	0.182	0.039	4.61	4.44 × 10^−6^
Vaspin	0.080	0.029	0.081	0.030	2.68	7.42 × 10^−3^

RMDQ, Roland-Morris Disability Questionnaire; SE, standard error; ECW, extracellular water; At the initial stage of the study, the following independent variables were tested in stepwise-forward manners: age, sex, WHR, BCM/WT, ECW, vaspin; all quantitative variables were standardized prior to statistical analysis.

**Table 5:** Summary of the series quasi- and variance component analyses of the studied phenotypes. Additive genetic components as estimated in most parsimonious models are shown.

Variable	Additive Genetic	*p*-Value
Vaspin	0.66 ± 0.08	2.64 × 10^−6^
ECW	0.42± 0.06	0.0005
LBP-sciatica	0.48 ± 0.18	NS
LBP-duration	0.31 ± 0.36	NS
LBP-severity	0.54 ± 0.13	NS
LBP-disability	0.19 ± 0.02	2.42 × 10^−5^

ECW, extracellular water; NS, non-significant.

## Appendix A

**Table A1:** Basic descriptive statistics of anthropometric measurements and plasma levels of studied soluble markers according to gender.

Variables	Gender	*N*	X ± SE	95%CI	*p*
Age (y)	M	489	42.80 ± 0.62	(41.58, 44.05)	0.651
	F	589	43.20 ± 0.56	(42.09, 44.31)	
BMI (kg/m^2^)	M	486	27.53 ± 0.19	(27.15, 27.92)	0.00027
	F	589	28.72 ± 0.24	(28.23, 29.21)	
WHR	M	483	0.92 ± 0.003	(0.92, 0.93)	<0.001
	F	579	0.88 ± 0.004	(0.87, 0.88)	
FM/WT	M	481	0.25 ± 0.002	(0.25, 0.26)	<0.001
	F	575	0.36 ± 0.003	(0.36, 0.37)	
SMM/WT	M	478	0.37 ± 0.002	(0.37, 0.38)	<0.001
	F	573	0.27 ± 0.001	(0.27,0.28)	
BCM/WT	M	481	0.39 ± 0.002	(0.39, 0.40)	<0.001
	F	575	0.31 ± 0.002	(0.31, 0.32)	
ECW (L)	M	483	20.75 ± 0.15	(20.46, 21.05)	<0.001
	F	576	16.53 ± 0.10	(16.32, 16.74)	
Vaspin (pg/mL)	M	458	1603.59 ± 233.68	(1144.35, 2062.82)	0.0011 *
	F	547	1605.21 ± 210.53	1191.68, 2018.79)	
Adipsin (µg/mL)	M	480	1.30 ± 0.01	(1.27, 1.34)	0.054 *
	F	586	1.28 ± 0.01	(1.25, 1.32)	

Data are presented as mean ± standard errors with 95% confidence intervals; N, sample size; BMI, body mass index; WHR, waist-to-hip ratio; FM/WT, fat mass/weight ratio; SMM/WT, skeletal muscle mass/weight ratio; BCM/WT, body cell mass/weight ratio, ECW, extracellular water; *p* values were determined by one way ANOVA, * these variables were compared also by Man-Whitney test.

**Table A2:** Correlations between body composition parameters and plasma levels of soluble markers in the study sample, in men and women separately; males’ and females’ data are shown above and below the diagonal, respectively.

	AGE	BMI	WHR	FM/WT	SMM/WT	BCM/WT	ECW	Vaspin	Adipsin
AGE		0.28 **	0.56 **	0.29 **	−0.45 **	−0.53 **	0.32 **	NS	0.18 **
BMI	0.61 **		0.55 **	0.78 **	−0.78 **	−0.46 **	0.58 **	0.11 *	0.17 *
WHR	0.49 **	0.44 **		0.52 **	−0.58 **	−0.51 **	0.44 **	NS	0.12 **
FM/WT	0.53 **	0.87 **	0.41 **		−0.96 **	−0.75 **	0.49 **	NS	0.15 **
SMM/WT	−0.66 **	−0.81 **	−0.45 **	−0.94 **		0.70 **	−0.42 **	NS	−0.16 **
BCM/WT	−0.48 **	−0.63 **	−0.32 **	−0.83 **	0.75 **		−0.66 **	−0.10 *	−0.18 **
ECW	0.46 **	0.67 **	0.27 **	0.63 **	−0.46 **	-0.76 **		0.14 **	0.19 **
Vaspin	NS	NS	NS	NS	NS	NS	NS		NS
Adipsin	0.31 **	0.35 **	0.15 **	0.31 **	−0.29 **	−0.30 **	0.33 **	NS	

BMI, body mass index; WHR, waist-to-hip ratio; FM/WT, fat mass/weight ratio; SMM/WT, skeletal muscle mass/weight ratio; BCM/WT, body cell mass/weight ratio, ECW, extracellular water; Pearson correlation coefficients (r) are presented, ** < 0.01, * < 0.05, NS -non-significant-*p* > 0.05.

**Table A3:** Comparative analysis of body composition parameters and plasma levels of soluble markers in individuals with LBP-related phenotypes according to gender.

	LBP-Sciatica			LBP-Duration			LBP-Severity		
Covariate	Male, *N*= 180	Female, *N*= 268	*p*	Male, *N*= 132	Female, *N* = 195	*p*	Male, *N* = 195	Female, *N* = 150	*p*
Age (y)	46.97 ± 0.95	47.79 ± 0.79	5	49.49 ± 1.10	49.87 ± 0.88	NS	46.94 ± 1.33	48.30 ± 1.13	NS
BMI (kg/m^2^)	27.89 ± 0.32	30.40 ± 0.35	5	28.12 ± 0.39	31.02 ± 0.41	5	28.03 ± 0.46	30.86 ± 0.48	5
WHR	0.94 ± 0.005	0.90 ± 0.005	5	0.95 ± 0.006	0.91 ± 0.007	4	0.94 ± 0.008	0.92 ± 0.009	NS
FM/WT	0.25 ± 0.004	0.38 ± 0.004	4	0.25 ± 0.005	0.39 ± 0.004	4	0.25 ± 0.006	0.39 ± 0.006	4
SMM/WT	0.37 ± 0.003	0.26 ± 0.002	4	0.37 ± 0.004	0.26 ± 0.002	4	0.37 ± 0.004	0.26 ± 0.003	4
BCM/WT	0.39 ± 0.004	0.29 ± 0.003	4	0.38 ± 0.005	0.29 ± 0.003	4	0.38 ± 0.005	0.29 ± 0.004	4
ECW (L)	21.54 ± 0.27	17.31 ± 0.16	4	21.83 ± 0.33	17.64 ± 0.19	4	22.11 ± 0.39	17.40 ± 0.25	4
Vaspin (pg/mL)	6.00 ± 0.09	6.21 ± 0.08	NS	6.00 ± 0.10	6.20 ± 0.09	NS	6.24 ± 0.16	6.12 ± 0.10	NS
Adipsin (µg/mL)	0.22 ± 0.01	0.26 ± 0.01	NS	0.24 ± 0.02	0.27 ± 0.02	NS	0.23 ± 0.02	0.26 ± 0.01	NS

Data presented as mean, standard errors; N, sample size; BMI, body mass index; WHR, waist-to-hip ratio; FM/WT, fat mass/weight ratio; SMM/WT, skeletal muscle mass/weight ratio; BCM/WT, body cell mass/weight ratio, ECW, extracellular water; *p* shows significance levels achieved upon comparison of each LBP-related phenotype by sex, 1 ≤0.05; 2≤0.01; 3≤0.001; 4≤0.0001; 5 ≤0.00001, NS -non-significant; Adipokines’ circulating levels were transformed to approximate normality prior to analysis.

## References

[1] Lamont L.A., Tranquilli W.J., Grimm K.A. (2000). Physiology of Pain. Vet. Clin. North Am. Small Anim. Pr..

[2] Hartvigsen J., Hancock M.J., Kongsted A., Louw Q., Ferreira M.L., Genevay S., Hoy D., Karppinen J., Pransky G., Sieper J. (2018). What low back pain is and why we need to pay attention. Lancet.

[3] Ignasiak D., Valenzuela W., Reyes M., Ferguson S.J. (2018). The effect of muscle ageing and sarcopenia on spinal segmental loads. Eur. Spine J..

[4] Kim W.J., Kim K.J., Song D.G., Lee J.S., Park K.Y., Lee J.W., Chang S.H., Choy W.S. (2020). Sarcopenia and Back Muscle Degeneration as Risk Factors for Back Pain: A Comparative Study. Asian Spine J..

[5] Sakai Y., Matsui H., Ito S., Hida T., Ito K., Koshimizu H., Harada A. (2017). Sarcopenia in elderly patients with chronic low back pain. Osteoporos. Sarcopenia.

[6] Tarabeih N., Shalata A., Trofimov S., Kalinkovich A., Livshits G. (2019). Growth and differentiation factor 15 is a biomarker for low back pain-associated disability. Cytokine.

[7] Risbud M.V., Shapiro I.M. (2014). Role of cytokines in intervertebral disc degeneration: Pain and disc content. Nat. Rev. Rheumatol..

[8] Rider S.M., Mizuno S., Kang J.D. (2019). Molecular mechanisms of intervertebral disc degeneration. Spine Surg. Relat. Res..

[9] Molinos M., Almeida C.R., Caldeira J., Cunha C., Gonçalves R.M., Barbosa M.A. (2015). Inflammation in intervertebral disc degeneration and regeneration. J. R. Soc. Interface.

[10] Navone S.E., Marfia G., Giannoni A., Beretta M., Guarnaccia L., Gualtierotti R., Nicoli D., Rampini P., Campanella R. (2017). Inflammatory mediators and signalling pathways controlling intervertebral disc degeneration. Histol. Histopathol..

[11] Morris P., Ali K., Merritt M., Pelletier J., Macedo L.G. (2020). A systematic review of the role of inflammatory biomarkers in acute, subacute and chronic non-specific low back pain. BMC Musculoskelet. Disord..

[12] Hider S.L., Konstantinou K., Hay E.M., Glossop J., Mattey D.L. (2019). Inflammatory biomarkers do not distinguish between patients with sciatica and referred leg pain within a primary care population: Results from a nested study within the ATLAS cohort. BMC Musculoskelet. Disord..

[13] Ruiz-Fernández C., Francisco V., Pino J., Mera A., González-Gay M.A., Gómez R., Lago F., Gualillo O. (2019). Molecular Relationships among Obesity, Inflammation and Intervertebral Disc Degeneration: Are Adipokines the Common Link?. Int. J. Mol. Sci..

[14] Hashem L.E., Roffey D.M., Alfasi A.M., Papineau G.D., Wai D.C., Phan P., Kingwell S.P., Wai E.K. (2018). Exploration of the inter-relationships between obesity, physical inactivity, inflammation, and low back pain. Spine (Phila. Pa. 1976).

[15] Ferreira P.H., Beckenkamp P., Maher C.G., Hopper J.L., Ferreira M.L. (2013). Nature or nurture in low back pain? Results of a systematic review of studies based on twin samples. Eur. J. Pain.

[16] Doury-Panchout F., Metivier J.-C., Nardoux J., Fouquet B. (2017). Visceral obesity and chronic pain: Effect of a 4-week rehabilitation program on adipokines and insulin resistance. J. Exerc. Rehabil..

[17] Bas S., Finckh A., Puskas G.J., Suva D., Hoffmeyer P., Gabay C., Lübbeke A. (2014). Adipokines correlate with pain in lower limb osteoarthritis: Different associations in hip and knee. Int. Orthop..

[18] Calvet J., Orellana C., Albiñana Giménez N., Berenguer-Llergo A., Caixàs A., García-Manrique M., Galisteo Lencastre C., Navarro N., Larrosa M., Gratacós J. (2018). Differential involvement of synovial adipokines in pain and physical function in female patients with knee osteoarthritis. A cross-sectional study. Osteoarthr. Cart..

[19] Sharma A. (2018). The Role of Adipokines in Intervertebral Disc Degeneration. Med. Sci..

[20] Neumann E., Junker S., Schett G., Frommer K., Müller-Ladner U. (2016). Adipokines in bone disease. Nat. Rev. Rheumatol..

[21] Shiri R., Solovieva S., Husgafvel-Pursiainen K., Taimela S., Saarikoski L.A., Huupponen R., Viikari J., Raitakari O.T., Viikari-Juntura E. (2008). The association between obesity and the prevalence of low back pain in young adults: The Cardiovascular Risk in Young Finns Study. Am. J. Epidemiol..

[22] Montagnana M., Fava C., Targher G., Franchini M., Danese E., Bonafini S., De Cata A., Salvagno G.L., Ruzzenente O., Guidi G.C. (2017). Plasma Leptin in Patients at Intermediate to High Cardiovascular Risk With and Without Type 2 Diabetes Mellitus. J. Clin. Lab. Anal..

[23] Valverde-Franco G., Tardif G., Mineau F., Paré F., Lussier B., Fahmi H., Pelletier J.-P., Martel-Pelletier J. (2018). High in vivo levels of adipsin lead to increased knee tissue degradation in osteoarthritis: Data from humans and animal models. Rheumatology.

[24] Brady S.R.E., Mousa A., Naderpoor N., de Courten M.P.J., Cicuttini F., de Courten B. (2018). Adipsin Concentrations Are Associated with Back Pain Independently of Adiposity in Overweight or Obese Adults. Front. Physiol..

[25] Körner A., Neef M., Friebe D., Erbs S., Kratzsch J., Dittrich K., Blüher S., Kapellen T.M., Kovacs P., Stumvoll M. (2011). Vaspin is related to gender, puberty and deteriorating insulin sensitivity in children. Int. J. Obes..

[26] Goktas Z., Owens S., Boylan M., Syn D., Shen C.-L., Reed D.B., San Francisco S., Wang S. (2013). Associations between Tissue Visfatin/Nicotinamide, Phosphoribosyltransferase (Nampt), Retinol Binding Protein-4, and Vaspin Concentrations and Insulin Resistance in Morbidly Obese Subjects. Mediat. Inflamm..

[27] Feng R., Li Y., Wang C., Luo C., Liu L., Chuo F., Li Q., Sun C. (2014). Higher vaspin levels in subjects with obesity and type 2 diabetes mellitus: A meta-analysis. Diabetes Res. Clin. Pr..

[28] Nicholson T., Church C., Tsintzas K., Jones R., Breen L., Davis E.T., Baker D.J., Jones S.W. (2019). Vaspin promotes insulin sensitivity in elderly muscle and is upregulated in obesity. J. Endocrinol..

[29] Wang H., Chen F., Li J., Wang Y., Jiang C., Wang Y., Zhang M., Xu J. (2020). Vaspin antagonizes high fat-induced bone loss in rats and promotes osteoblastic differentiation in primary rat osteoblasts through Smad-Runx2 signaling pathway. Nutr. Metab..

[30] Kalichman L., Carmeli E., Been E. (2017). The Association between Imaging Parameters of the Paraspinal Muscles, Spinal Degeneration, and Low Back Pain. Biomed Res. Int..

[31] Okamoto C.S., Dunn A.S., Green B.N., Formolo L.R., Chicoine D. (2017). Correlation of Body Composition and Low Back Pain Severity in a Cross-Section of US Veterans. J. Manip. Physiol..

[32] Walsh T.P., Arnold J.B., Evans A.M., Yaxley A., Damarell R.A., Shanahan E.M. (2018). The association between body fat and musculoskeletal pain: A systematic review and meta-analysis. BMC Musculoskelet Disord..

[33] Endo T., Abe T., Akai K., Kijima T., Takeda M., Yamasaki M., Isomura M., Nabika T., Yano S. (2019). Height loss but not body composition is related to low back pain in community-dwelling elderlies: Shimane CoHRE study. BMC Musculoskelet Disord..

[34] Heuch I., Heuch I., Hagen K., Zwart J.A. (2015). A comparison of anthropometric measures for assessing the association between body size and risk of chronic low back pain: The HUNT study. PLoS ONE.

[35] Hussain S.M., Urquhart D.M., Wang Y., Shaw J.E., Magliano D.J., Wluka A.E., Cicuttini F.M. (2017). Fat mass and fat distribution are associated with low back pain intensity and disability: Results from a cohort study. Arthritis Res..

[36] Brady S.R.E., Urquhart D.M., Hussain S.M., Teichtahl A., Wang Y., Wluka A.E., Cicuttini F. (2019). High baseline fat mass, but not lean tissue mass, is associated with high intensity low back pain and disability in community-based adults. Arthritis Res..

[37] Livshits G., Cohen Z., Higla O., Yakovenko K. (2001). Familial history, age and smoking are important risk factors for disc degeneration disease in Arabic pedigrees. Eur. J. Epidemiol..

[38] Smedley J., Inskip H., Cooper C., Coggon D. (1998). Natural history of low back pain. A longitudinal study in nurses. Spine.

[39] Chiarotto A., Maxwell L.J., Terwee C.B., Wells G.A., Tugwell P., Ostelo R.W. (2016). Roland-Morris Disability Questionnaire and Oswestry Disability Index: Which Has Better Measurement Properties for Measuring Physical Functioning in Nonspecific Low Back Pain? Systematic Review and Meta-Analysis. Phys. Ther..

[40] Roland M., Fairbank J. (2000). The Roland-Morris Disability Questionnaire and the Oswestry Disability Questionnaire. Spine.

[41] McManus M.L., Churchwell K.B., Strange K. (1995). Regulation of cell volume in health and disease. N. Engl. J. Med..

[42] Malkin I., Williams F.M.K., LaChance G., Spector T., MacGregor A.J., Livshits G. (2014). Low back and common widespread pain share common genetic determinants. Ann. Hum. Genet..

[43] Falconer D.S., Douglas S., Mackay T.F.C. (1996). Introduction to Quantitative Genetics.

[44] Fourney D.R., Andersson G., Arnold P.M., Dettori J., Cahana A., Fehlings M.G., Norvell D., Samartzis D., Chapman J.R. (2011). Chronic Low Back Pain. Spine.

[45] Heiker J.T. (2014). Vaspin (serpinA12) in obesity, insulin resistance, and inflammation. J. Pept. Sci..

[46] Dimova R., Tankova T. (2015). The Role of Vaspin in the Development of Metabolic and Glucose Tolerance Disorders and Atherosclerosis. Biomed. Res. Int..

[47] Liu Y., Xu F., Pei H.-X., Zhu X., Lin X., Song C.-Y., Liang Q.-H., Liao E.-Y., Yuan L.-Q. (2016). Vaspin regulates the osteogenic differentiation of MC3T3-E1 through the PI3K-Akt/miR-34c loop. Sci. Rep..

[48] Bao J.-P., Jiang L.-F., Chen W.-P., Hu P.-F., Wu L.-D. (2014). Expression of vaspin in the joint and the levels in the serum and synovial fluid of patients with osteoarthritis. Int. J. Clin. Exp. Med..

[49] Jung C.H., Lee W.J., Hwang J.Y., Seol S.M., Kim Y.M., La Lee Y., Park J.-Y. (2011). Vaspin protects vascular endothelial cells against free fatty acid-induced apoptosis through a phosphatidylinositol 3-kinase/Akt pathway. Biochem. Biophys. Res. Commun..

[50] Bao J., Xu L., Ran J., Xiong Y., Wu L. (2017). Vaspin prevents leptin-induced inflammation and catabolism by inhibiting the activation of nuclear factor-κB in rat chondrocytes. Mol. Med. Rep..

[51] Li X., Ke X., Li Z., Li B. (2019). Vaspin prevents myocardial injury in rats model of diabetic cardiomyopathy by enhancing autophagy and inhibiting inflammation. Biochem. Biophys. Res. Commun..

[52] Youn B.-S., Kloting N., Kratzsch J., Lee N., Park J.W., Song E.-S., Ruschke K., Oberbach A., Fasshauer M., Stumvoll M. (2008). Serum Vaspin Concentrations in Human Obesity and Type 2 Diabetes. Diabetes.

[53] Teshigawara S., Wada J., Hida K., Nakatsuka A., Eguchi J., Murakami K., Kanzaki M., Inoue K., Terami T., Katayama A. (2012). Serum Vaspin Concentrations Are Closely Related to Insulin Resistance, and rs77060950 at *SERPINA12* Genetically Defines Distinct Group with Higher Serum Levels in Japanese Population. J. Clin. Endocrinol. Metab..

[54] Goode A.P., Carey T.S., Jordan J.M. (2013). Low Back Pain and Lumbar Spine Osteoarthritis: How Are They Related?. Curr. Rheumatol. Rep..

[55] Matsuda M., Huh Y., Ji R.-R. (2019). Roles of inflammation, neurogenic inflammation, and neuroinflammation in pain. J. Anesth..

[56] Brocker C., Thompson D.C., Vasiliou V. (2012). The role of hyperosmotic stress in inflammation and disease. Biomol. Concepts.

[57] Vergroesen P.-P.A., Kingma I., Emanuel K.S., Hoogendoorn R.J.W., Welting T.J., van Royen B.J., van Dieën J.H., Smit T.H. (2015). Mechanics and biology in intervertebral disc degeneration: A vicious circle. Osteoarthr. Cart..

[58] Stookey J.D., Barclay D., Arieff A., Popkin B.M. (2007). The altered fluid distribution in obesity may reflect plasma hypertonicity. Eur. J. Clin. Nutr..

[59] Yoon H.-J., You S., Yoo S.-A., Kim N.-H., Kwon H.M., Yoon C.-H., Cho C.-S., Hwang D., Kim W.-U. (2011). NF-AT5 is a critical regulator of inflammatory arthritis. Arthritis Rheum..

[60] Serra-Prat M., Lorenzo I., Palomera E., Yébenes J., Campins L., Cabré M. (2019). Intracellular Water Content in Lean Mass is Associated with Muscle Strength, Functional Capacity, and Frailty in Community-Dwelling Elderly Individuals. A Cross-Sectional Study. Nutrients.

[61] Yamada Y., Yoshida T., Yokoyama K., Watanabe Y., Miyake M., Yamagata E., Yamada M., Kimura M. (2016). The Extracellular to Intracellular Water Ratio in Upper Legs is Negatively Associated With Skeletal Muscle Strength and Gait Speed in Older People. J. Gerontol. Ser. A Biol. Sci. Med. Sci..

[62] Serra-Prat M., Lorenzo I., Palomera E., Ramírez S., Yébenes J.C. (2019). Total Body Water and Intracellular Water Relationships with Muscle Strength, Frailty and Functional Performance in an Elderly Population. A Cross-Sectional Study. J. Nutr. Heal. Aging.

[63] Abdel Ghany S.M., Sayed A.A., El-Deek S.E.M., ElBadre H.M., Dahpy M.A., Saleh M.A., Sharaf El-Deen H., Mustafa M.H. (2017). Obesity risk prediction among women of Upper Egypt: The impact of serum vaspin and vaspin rs2236242 gene polymorphism. Gene.

[64] Suliga E., Kozieł D., Cieśla E., Rębak D., Wawszczak M., Adamus-Białek W., Naszydłowska E., Piechowska A., Głuszek S. (2019). Associations between vaspin rs2236242 gene polymorphism, walking time and the risk of metabolic syndrome. Balk. J. Med. Genet..

[65] Alnory A., Gad H., Hegazy G., Shaker O. (2016). The association of vaspin rs2236242 and leptin rs7799039 polymorphism with metabolic syndrome in Egyptian women. Turk. J. Med. Sci..

[66] Hanisch D., Dittmar M., Höhler T., Alt K.W. (2004). Contribution of genetic and environmental factors to variation in body compartments—A twin study in adults. Anthropol. Anzeiger.

[67] Lippi G., Dagostino C., Buonocore R., Aloe R., Bonaguri C., Fanelli G., Allegri M. (2017). The serum concentrations of leptin and MCP-1 independently predict low back pain duration. Clin. Chem. Lab. Med..

[68] Weber K.T., Satoh S., Alipui D.O., Virojanapa J., Levine M., Sison C., Quraishi S., Bloom O., Chahine N.O. (2015). Exploratory study for identifying systemic biomarkers that correlate with pain response in patients with intervertebral disc disorders. Immunol. Res..

[69] Li Y., Liu J., Liu Z.-Z., Duan D.-P. (2016). Inflammation in low back pain may be detected from the peripheral blood: Suggestions for biomarker. Biosci. Rep..

